# Editorial: Mathematical problems in physical fluid dynamics: part II

**DOI:** 10.1098/rsta.2021.0057

**Published:** 2022-06-27

**Authors:** D. Goluskin, B. Protas, J.-L. Thiffeault

**Affiliations:** ^1^ Department of Mathematics and Statistics, University of Victoria, Victoria, British Columbia, Canada; ^2^ Department of Mathematics and Statistics, McMaster University, Hamilton, Ontario, Canada; ^3^ Department of Mathematics, University of Wisconsin-Madison, Madison, WI, USA

**Keywords:** turbulence, transport, convection, mixing, vortex dynamics, *a priori* bounds

## Abstract

Fluid dynamics is a research area lying at the crossroads of physics and applied mathematics with an ever-expanding range of applications in natural sciences and engineering. However, despite decades of concerted research efforts, this area abounds with many fundamental questions that still remain unanswered. At the heart of these problems often lie mathematical models, usually in the form of partial differential equations, and many of the open questions concern the validity of these models and what can be learned from them about the physical problems. In recent years, significant progress has been made on a number of open problems in this area, often using approaches that transcend traditional discipline boundaries by combining modern methods of modelling, computation and mathematical analysis. The two-part theme issue aims to represent the breadth of these approaches, focusing on problems that are mathematical in nature but help to understand aspects of real physical importance such as fluid dynamical stability, transport, mixing, dissipation and vortex dynamics.

This article is part of the theme issue ‘Mathematical problems in physical fluid dynamics (part 2)’.

We refer the reader to the introduction of Part I of the theme issue for a discussion of its overarching topics and broader relevance. The main topics of Part II of the theme issue are vortex dynamics and turbulence, with several interstitial areas included as well. Gomez-Serrano *et al*. [1] find new relative equilibria involving vortex sheets that bifurcate from circular vortex sheets with constant magnitude. Ceci & Seis [2] study the evolution of two-dimensional Euler flows with highly concentrated vorticity and establish new connections to point-vortex dynamics. Equations of motion for two-dimensional compressible point vortices are derived in the limit of small Mach number by Llewelyn Smith *et al*. [3]. Ohkitani [4] constructs and analyses, using asymptotic techniques, self-similar solutions to the one-dimensional Burgers equation and the two-dimensional and three-dimensional Navier–Stokes systems, which complement previously known solutions of this type. Solutions to the three-dimensional Euler equations are constructed by Bustamante [5] using continuous symmetries leading to new conditions for blow-up. Parente *et al*. [6] use a continuation method to find coherent solutions to the Navier–Stokes equations.

Turning to the contributions with especially close connections to turbulence, Tsuruhashi *et al*. [7] use direct numerical simulation to analyse the turbulent cascade in three-dimensional flows in terms of self-similar stretching of coherent vortices. Drivas [8] demonstrates that, under the assumption of anti-alignment of velocity increments, inertial dissipation provides a regularization mechanism for turbulence. New estimates for the energy dissipation in general eddy viscosity models for shear flows are established by Kean *et al*. [9]. Van Kan *et al*. [10] use an analysis based on geometric microcanonical ensembles to develop a statistical physics approach to study the evolution of the Fourier-truncated two-dimensional Euler system. Schorlepp *et al*. [11] employ the instanton formalism to identify extreme vorticity and strain events in stochastically forced three-dimensional Navier–Stokes flows. Gomé *et al*. [12] explore how splitting and extinction of localized turbulence in transitional shear flows can be modelled probabilistically using rare event methods. Simulations investigating whether the moist Boussinseq equations converge to the precipitating quasi-geostrophic equations in a particular parametric limit are carried out by Zhang *et al*. [13].

## Data Availability

This article has no additional data.
